# Effect of slow release nitrogenous fertilizers and biochar on growth, physiology, yield, and nitrogen use efficiency of sunflower under arid climate

**DOI:** 10.1007/s11356-022-19289-6

**Published:** 2022-03-09

**Authors:** Muhammad Waqar, Muhammad Habib-ur-Rahman, Muhammad Usama Hasnain, Shahid Iqbal, Abdul Ghaffar, Rashid Iqbal, Muhammad Iftikhar Hussain, Ayman EL Sabagh

**Affiliations:** 1 Department of Agronomy, Muhammad Nawaz Shareef University of Agriculture, Multan, Pakistan; 2 Crop Science Group, Institute of Crop Science and Resource Conservation (INRES), University of Bonn, Bonn, Germany; 3 Institute of Plant Breeding and Biotechnology, MNS University of Agriculture Multan, Multan, Pakistan; 4 Department of Agronomy, Faculty of Agriculture & Environment, The Islamia University of Bahawalpur, Bahawalpur, Pakistan; 5 Department of Plant Biology and Soil Science, Universidad de Vigo, Campus Lagoas Marcosende, 36310 Vigo, Spain; 6 Siirt University, Faculty of Agriculture, Department of Field Crops, Siirt, 56100 Turkey

**Keywords:** Loaded biochar, Neem-coated urea, Leaf photosynthetic rate, Stomatal conductance, Sulfur-coated urea

## Abstract

Sunflower plants need nitrogen consistently and in higher amount for optimum growth and development. However, nitrogen use efficiency (NUE) of sunflower crop is low due to various nitrogen (N) losses. Therefore, it is necessary to evaluate the advanced strategies to minimize N losses and also improve sunflower productivity under arid climatic conditions. A field trial was conducted with four slow release nitrogenous fertilizers [SRNF (bacterial, neem, and sulfur-coated urea and N loaded biochar)] and three N levels (100% = 148 kg N ha^−1^, 80% = 118 kg N ha^−1^, and 60% = 89 kg N ha^−1^) of recommended application (100%) for sunflower crop under arid climatic conditions. Results showed that neem-coated urea at 148 kg N ha^−1^ significantly enhanced crop growth rate (CGR) (19.16 g m^−2^ d^−1^) at 60–75 days after sowing (DAS); leaf area index (2.12, 3.62, 5.97, and 3.00) at 45, 60, 75, and 90 DAS; and total dry matter (14.27, 26.29, 122.67, 410, and 604.33 g m^−2^) at 30, 45, 60, 75, and 90 DAS. Furthermore, higher values of net leaf photosynthetic rate (25.2 µmol m^−2^ s^−1^), transpiration rate (3.66 mmol s^−1^), and leaf stomatal conductance (0.39 mol m^−2^ s^−1^) were recorded for the same treatment. Similarly, neem-coated urea produced maximum achene yield (2322 kg ha^−1^), biological yield (9000 kg ha^−1^), and harvest index (25.8%) of the sunflower crop. Among various N fertilizers, neem-coated urea showed maximum NUE (20.20 kg achene yield kg^−1^ N applied) in comparison to other slow release N fertilizers. Similarly, nitrogen increment N_60_ showed maximum NUE (22.40 kg grain yield kg^−1^ N applied) in comparison to N_80_ and N_100_. In conclusion, neem-coated urea with 100% and 80% of recommended N would be recommended for farmers to get better sunflower productivity with sustainable production and to reduce the environmental nitrogen losses.

## Introduction

Sunflower is an important and third-largest oilseed crop after soybean and rapeseed grown throughout the subtropical and temperate regions of the world (Demir et al. 2006; Usman et al. 2010; Adeleke and Babalola, 2020). It is also an emerging and third-largest oilseed crop after cotton and mustard cultivated on 151 thousand acres area with 33 thousand tons production of oil per annum in Pakistan (GOP, 2020–2021; Rahman et al. 2019). Globally, sunflower productivity has shown a decreasing trend during the past decade, mainly because of lower resource use efficiency, reduced nitrogen use efficiency (NUE), extreme climatic conditions, and increased pest infestations and diseases due to imbalanced (N) fertilizer application (Ozer et al. 2004; Nasim et al. 2017; Tovar et al. 2021; Rahman et al. 2021; Perveen et al. 2021).

Emerging economies of the globe are the major users of N fertilizers. However, the major part of nitrogen application lost to the soil solution in the form of nitrate leaching, denitrification, and ammonia volatilization due to harsh climatic conditions (Beig et al. 2010). Nitrogen application should be done according to the demand of crops (Slafer and Savin 2018; Ghafoor et al. 2021). Nitrogen is readily available into the soil solution, and its required amount at that time is also vital factor for nitrogen uptake (Lin et al. 2017; Shafqat et al. 2021). Considerable amount of nitrogen leads to contamination of the environment and many losses in economic return (Rahman et al. 2020). The lodging of crops due to the unwise application of nitrogen fertilizers leads to economic loss. The effectiveness of monotypic urea is lower due to the quick release of N from a granule in the soil. The quick-release of N caused soil water and air pollution, through the contamination of groundwater and higher emissions of greenhouse gases into the atmosphere. Nitrogen is a major plant nutrient that is the fundamental constituent of amino acids and is involved in the synthesis of protein molecules. Hence, N fertilization has important function in improving the metabolic phenomena leading to vegetative and reproductive growth ultimately crop production (Koutroubas et al. 2008; Zubillaga et al. 2002; Snyder and Tegeder 2021). Moreover, the N fertilization increases the N leaf content which has a strongly affirmative correlation with the process of photosynthesis (Hassan et al. 2020; Ashraf et al. 2019; Evans and Clarke 2019). Various investigations have reported that N supplementation has also beneficial effects on the growth, physiology, and yield-related traits of sunflower crop (Khaliq et al., 2008; Nasim et al. 2012; Al Hasnawi et al. 2020). However, plants are unable to get access 40–70% of N applied through simple urea because of speedy release of N in the environment from the simple urea (Beig et al. 2010). The losses from simple urea are maximum because of high solubility in the water leading to constraints for aquatic life and therefore also disturbs flora and fauna (Zhang et al. 2014; Naz and Sulaiman 2015; Smith & Siciliano 2015; Trinh et al. 2015). Hence, farmers are applying an additional quantity of N fertilizer which adversely affects the income of agricultural farms. Moreover, some previous investigations have predicted about 30–60% losses of simple urea and ultimately considerable reductions in crop growth and yield (Anggoro, 2011; Naz and Sulaiman 2015). To overcome this problem, slower release N fertilizers would not only distribute (N) efficiently under field conditions but also makes it safe for the environment (Thind et al. 2009; Min et al. 2019).

Biochar is gaining much interest to improve soil health traits, mitigate climate change, and ultimately sustain crop productivity (Ahmad et al. 2021). For instance, improvement in soil aeration, water, and nutrient retention ability and soil microbial activity had reported under arid climatic conditions (Atkinson et al. 2010; Chan et al. 2007; Lehmann et al. 2011; Diatta et al. 2020). It has also positive influence on the reduction of the N losses by controlling nitrate leaching and carbon sequestration in the soil layers (Kammann et al. 2011; Shafqat et al. 2019; Steiner et al. 2008). Cotton crop has showed higher nitrogen use efficiency with the application of biochar which is attributed to increased microbial activity and N cycling in the soils (Basso et al. 2013; Manzoor et al. 2021). Furthermore, it reduces pollutants from the environment and can be used as an ecofriendly alternative to synthetic fertilizers (Bashir et al. 2021; Ijaz et al. 2020). Slow release N fertilizers are also used to reduce the nitrogen losses as they slowly release nitrogen and stabilize it for efficient utilization (Cantarella et al. 2018; Manzoor et al. 2021). Hence, nitrogen losses can be reduced through biochar application (Mandal et al. 2016; Rasuli et al. 2021). Thus, nitrogen use efficiency can be improved with the wise use of SRNF in the sunflower crop production (Garcia et al. 2018; Perveen et al. 2021). Therefore, it is vital to study the effects of SRNF and biochar in improving nitrogen use efficiency and sunflower achene yield. Hence, the main objectives of the field experiment were to study the effect of SRNF and biochar for sunflower growth, physiology, yield-related traits, and NUE under arid climatic conditions.

## Materials and methods

### Environmental conditions of the study site

**Fig. 1:**
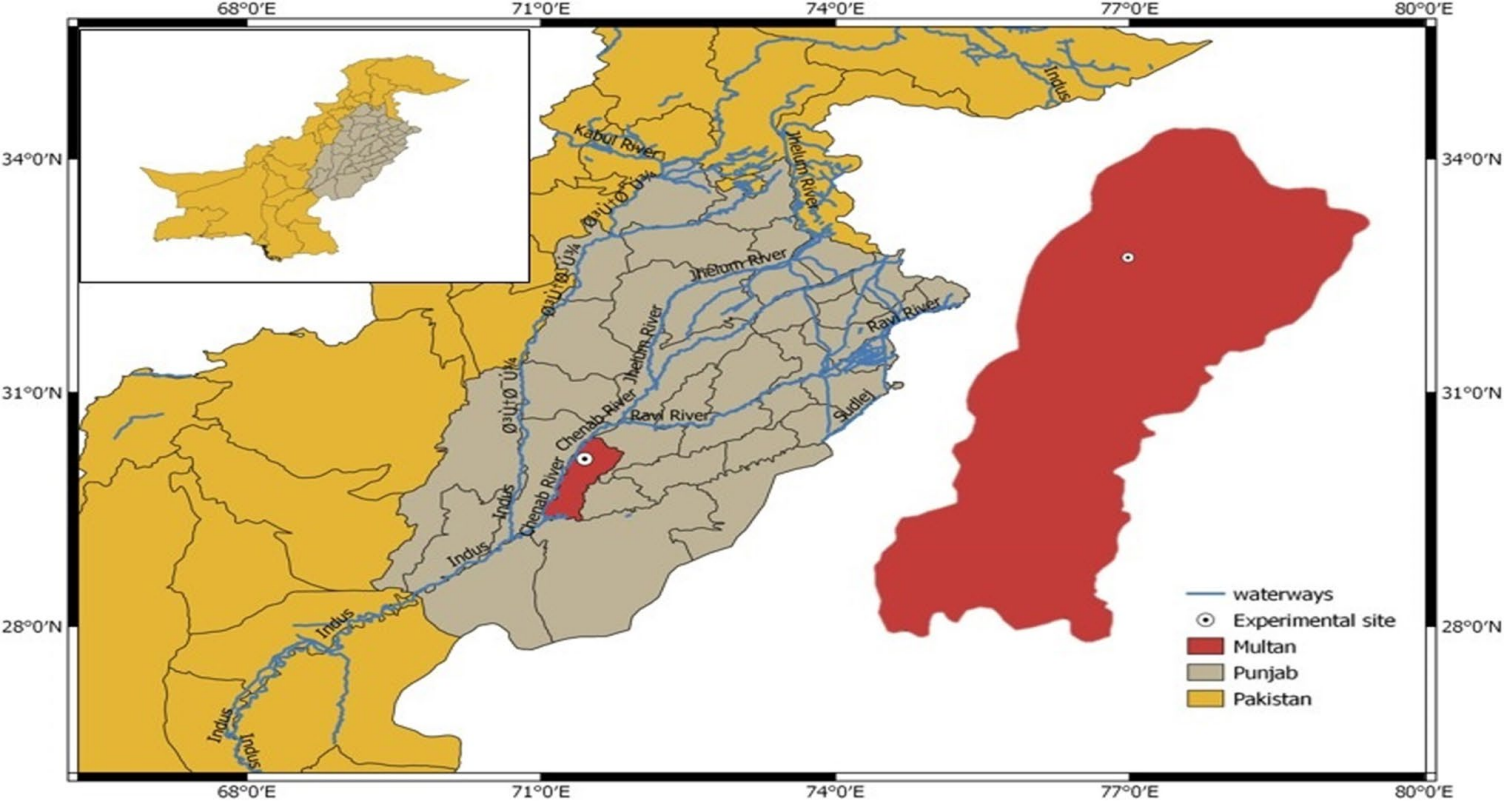
The locality of the field study in an arid climatic region of South Punjab, Pakistan

**Fig. 2:**
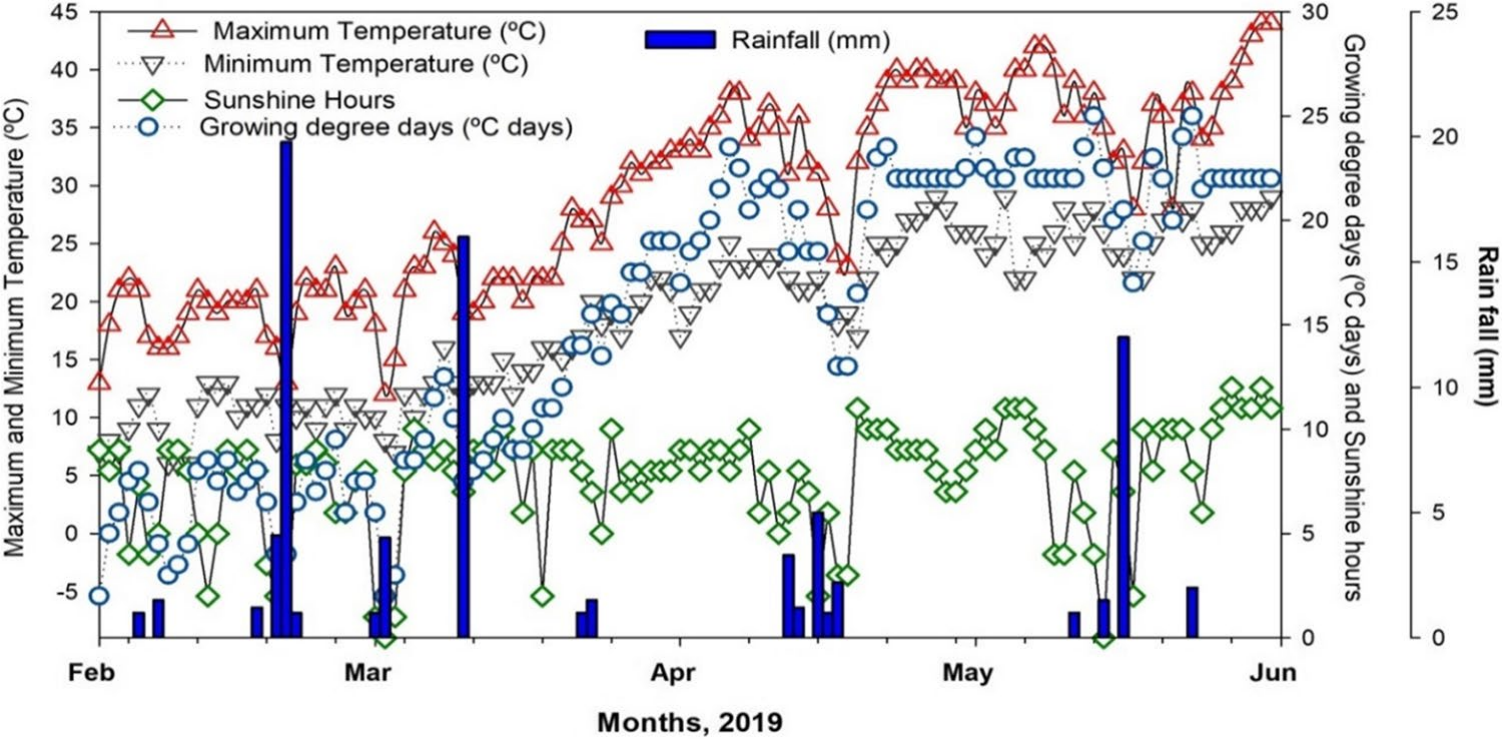
Daily data of weather variables at study site from February to June during crop growing period

The present work was executed at the research farm of MNS-University of Agriculture Multan, Pakistan (30°15 North, 71°52 East) in spring season 2019 under irrigated conditions (Fig. 1). The altitude of the current field was about 178 m above sea level. The study site falls under the country’s arid subtropical region comprising mean temperature of 32.6 °C and mean precipitation of 186.8 mm per annum. The diurnal variations observed in the study area fluctuated, with average air temperature varying from 26 to 49 °C and 4 to 23 °C throughout summer and winter periods, respectively. The maximum rainfall was recorded during the monsoon season. The regular values of minimum (Tmin), maximum (Tmax), sunshine hours, and the growing degree days (GDDs) (above a specific threshold temperature and rainfall) observed at automatic weather station installed at the experimental site throughout the study period are presented in Fig. 2. Weather conditions were too hot during summer month especially in May and June. The mean maximum temperature was about 45 °C during June, and the mean minimum temperature was observed about 13 °C in February (Fig. 2).

The research area was located between the rivers Sutlej (having water only under flood conditions since the 1960s) in the district Bahawalpur and the River Chenab (flowing freely throughout the year) in district Muzaffar Garh. The soil was silt loam in texture, river alluvium, brown, moderately calcareous, weakly structured, and hyperthermic. Fluventic Haplocambic, ochric epipedon, and cambic subsurface horizons were present (Miami Soil Series). The soil was low in organic matter (0.78%) which varied between different soil horizons (0.40–0.10%) and was deficient in organic N (0.05%), available phosphorus (7.90 mg kg^−1^), and available potassium (2.40 mg kg^−1^) in the upper layer (≤ 30 cm).

### Field experimental design and treatments

The experimental treatment is consisted of four different (N) sources. Plant growth-promoting bacteria (PGPBs) consisted of 46% N, neem-coated urea (NCU) consisted of 36% N, and sulfur-coated urea (SCU) consisted of 36% N were provided by Jaffer Agro-Services, Engro Fertilizers, and SAFI Chemicals and Fertilizers Limited, Pakistan, respectively. Biochar loaded urea and three different nitrogen levels (100% = 148 kg N ha^−1^, 80% = 118 kg N ha^−1^, and 60% = 89 kg N ha^−1^) of recommended dose (100%) were used in current study for sunflower crop as N100 (148 kg ha^-1^) is generally recommended by research institute for sunflower production in the region. Physicochemical properties of biochar used can be found in Khan et al. (2021). The experiment was done under field conditions using RCBD comprising four (4) replications and with a plot’s size of 5 × 4 m^2^ with leaving an appropriate border on both sides.

### Crop husbandry and crop management operations and practices

Sunflower genotype (Hysun-33) was used for this experiment, which is mostly adopted at farmer fields due to its better performance and adaptability under arid to semi-arid climatic conditions of the region. The experimental soil was ploughed two times followed by planking and leveling. Sowing was done on ridges. Ridger was tilled to prepare proper ridges (75 cm apart), and seed dibbling were done 25 cm apart at a 5-cm depth in the soil. Five irrigations were applied at 20-day intervals following weather conditions of the experimental site. Infestation of weeds was controlled through applying pre-emergence herbicides (Pendimethalin). All other recommended and optimum best crop management practices were adopted (Ahmad et al. 2020).

### Data recording and collection protocols

#### Growth parameters

Various growth parameters, LAI (leaf area index), TDM (total dry matter), and CGR (crop growth rate), were computed using their standard procedures and protocols. Watson proposed a formula to find LAI for the distinct samples (Watson, 1947). This formula was used to measure the LAI.

1 $$LAI=\frac{Leaf\,area}{Land\,area}$$

To estimate and assess the total dry matter, crop sample was cut down from one meter square which was started at 30 days after sowing and carried out at 15-day interval till harvesting of the sunflower crop. Fresh weight was taken from each part of the plants (stems, leaves) which was measured through weighing balance. Initially, soil samples dried at room temperature and then were oven-dried at 70 ℃. CGR was estimated as (W_2_ − W_1_) / (T_2_ − T_1_) (Lehmann et al. 2011) in which *W*_1_ and *W*_2_ indicate dry weights harvested at *T*_1_ and *T*_2_ time, respectively.

#### Physiological and yield related attributes

The net photosynthetic rates, net stomatal conductance, and net transpiration rate were recorded at the 70 days after sowing (full canopy point) by using CIRAS instrument of five selected plants from each treatment at maturity. CIRAS is a portable photosynthesis instrument which is abbreviated as CubeSat Infrared Atmospheric Sounder and produced by Dr. Keith J. Parkinson in 1984. A meter rod was used to measure the plant height at maturity from five tagged plants from each experimental unit, and their mean was calculated. Head diameter was estimated by Vernier caliper. The harvested crop was tied into bundles and after that sundried for 1 week in particular plots. Then crop was manually harvested, sun-dried, and threshed. Achene yield was calculated in kg ha^−1^ with the use of a weighing balance after threshing. One thousand achene were counted from each treatment, and weight was noted after weighing on electric balance. From every experimental unit, five heads were selected, and achenes were counted and their mean were calculated determined. Sunflower biomass was noted for each treatment by using a weighing balance.

#### Determination of nitrogen use efficiency and soil available nitrogen

NUE was measured by partial factor productivity (PFP) as well as nutrient balance in our study. The following formula was used to measure NUE. PFP was estimated by dividing achene yield to per kg applied nitrogen (Perveen et al. 2021).

2 $$\mathrm{PFP}\left(\mathrm{kg\;achene\;yield}/\mathrm{kg\;N\;applied}\right)=\mathrm{Achene\;yield}/\mathrm{N\;applied}$$

Soil auger was used to collect the sample from each experimental treatment, subsequent to the application of three doses of N. Then, samples collected from each experimental unit to the depths of 0–15 and 15–30 cm were analyzed. Standard procedures and protocol of alkaline permanganate was used for the determination of soil available (N) (SubbaiahV and Asija 1956).

### Growing degree days (◦C days) estimation for sunflower

For the determination of accumulated growing degree days [DD (°C days)], both the daily maximum temperature (Tmax) and the daily minimum temperature (Tmin) were used for estimation of DDs (Eq. 3) for the sunflower crop.

3 $$\mathrm{DD}(^\circ \mathrm{C days})={\sum }_{i=dh}^{i=ds}\left[\left\{\frac{Tmax+Tmin}{2}\right\}-TT\right]$$

where DD (°C days) stands for accumulative degree days for definite phenophase, “*ds*,” “*dh*,” and TT stands for date of sowing and harvest and threshold temperature, respectively, which was considered at 8 °C for sunflower to calculate the thermal time (Rahman et al. 2018; Ahmad et al. 2020). However, DD is measured as zero digit in the cases when [(Tmax + Tmin)/2] < TT, or [(Tmax + Tmin)/2] = TT.

### Statistical analysis

All the responsive observations were analyzed using analysis of variance technique (Steel et al. 1997;) via SAS version 9.4 (SAS Institute, Cary, NC 2013). The effect of SRNF, different N levels, and their interactive effect for all responsive observations was found by applying GLM model. The comparison of the experimental treatment means was carried out using LSD (least significant difference) test at *p* ≤ 0.05.

## Results

### Physiological parameters of sunflower crop under arid climatic conditions

The SRNF and N levels significantly affected the physiological traits (leaf chlorophyll content, net photosynthesis rate, stomatal conductance, and transpiration rate) of sunflower crop. However, interactive effect of different SRNF with N levels was not found to be significant for all responsive physiological parameters (Table 2). Sunflower crop showed the highest net leaf photosynthetic rate (26.8 µmol m^−2^ s^−1^) with neem-coated urea application which was at par when sunflower crop was fertilized with the loaded N biochar. The highest net photosynthesis rate (25.2 µmol m^−2^ s^−1^) was recorded when crop was fertilized at high N increment (148 kg ha^−1^) which was at par when sunflower crop fertilized with increment of N_80_ (118 kg ha^−1^) (Table 2). Similarly, sunflower crop showed the highest net transpiration rate (3.50 µmol m^−2^ s^−1^) with the application of neem-coated urea which was found at par when sunflower crop was fertilized with the loaded biochar and bacterial coated urea. Furthermore, sunflower crop fertilized with (N) increment N_100_ (148 kg ha^−1^) showed the highest net transpiration rate (3.66 µmol m^−2^ s^−1^). Sunflower crop showed highest leaf stomatal conductance (0.39 mmol m^−2^ s^−1^) with the application of neem-coated urea which was at par with the loaded biochar and bacterial coated urea. Moreover, sunflower crop fertilized with (N) increment N_100_ (148 kg ha^−1^) showed the highest leaf stomatal conductance (0.39 mmol m^−2^ s^−1^) (Table 2). Sunflower crop showed the highest chlorophyll content (59.6 spade value) with neem-coated urea which was at par with the loaded biochar and bacterial coated urea. Moreover, sunflower crop fertilized with (N) increment N_100_ (148 kg ha^−1^) also showed the highest chlorophyll content (60.6 spade value) (Table 1).

**Table 1:** Effect of different slow release nitrogen fertilizer (SRNF) and nitrogen increments on the leaf physiological parameters of sunflower crop under arid climatic conditions

Treatments	Net photosynthetic rate (µmol m^−2^ s^−1^)	Net transpiration rate (µmol m^−2^ s^−1^)	Stomatal conductance (mmol m^−2^ s^−1^)	Chlorophyll content (Spade value)
Slow release (N) fertilizers				
Neem-coated urea	26.8 a	3.50 a	0.39 a	59.6 a
Bacterial coated urea	25.7 c	3.43 a	0.37 a	58.8 ab
Sulfur-coated urea	21.1 d	2.69 b	0.29 b	58.5 b
Loaded biochar	26.3 b	3.50 a	0.38 a	59.3 ab
LSD (*p* ≤ 0.05)	0.3623	0.1875	0.0311	0.90
N increments				
N_100_ = 148 kg ha^−1^	25.2 a	3.66 a	0.39 a	60.9 a
N_80_ = 118 kg ha^−1^	25.1 a	3.32 b	0.35 b	58.9 b
N_60_ = 89 kg ha^−1^	24.6 b	2.87 c	0.34 b	57.4 c
LSD (*p* ≤ 0.05)	0.2839	0.1469	0.0243	0.71
SRNF	**	**	**	**
NI	**	**	**	**
SRNF × NI	NS	NS	NS	NS

*SRNF* slow release (N) fertilizers; *NI* (N) increments; **significant at *p* ≤ 0.01; *significant at *p* ≤ 0.05; *NS* non-significant at *p* ≤ 0.05.

### Growth parameters (LAI, CGR, and TDM) of sunflower crop under arid climatic conditions

**Fig. 3:**
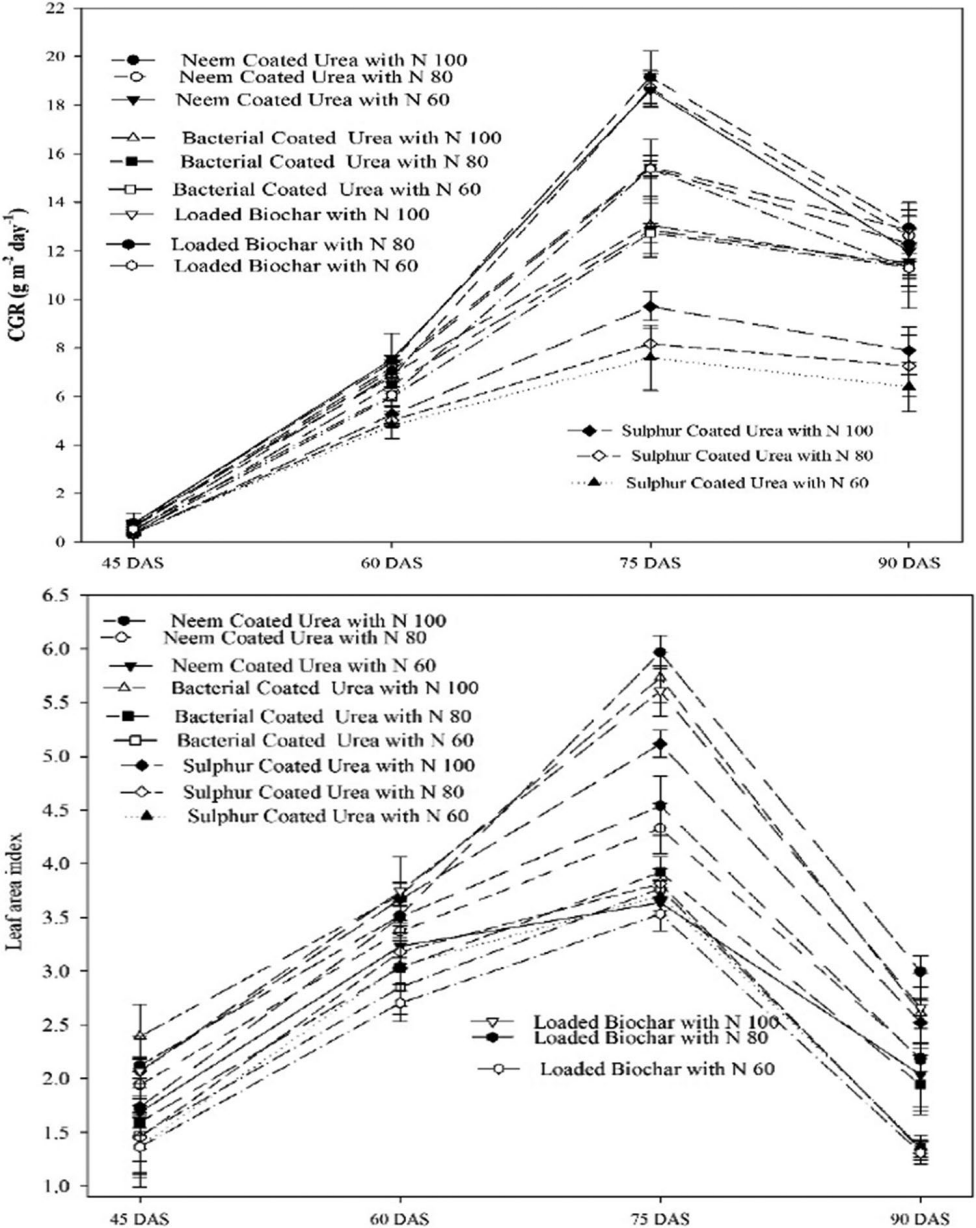
Effect of slow release nitrogen fertilizer (SRNF) and nitrogen levels on the crop growth rate and leaf area index at different crop critical growth stages of sunflower crop under arid climatic conditions

**Fig. 4:**
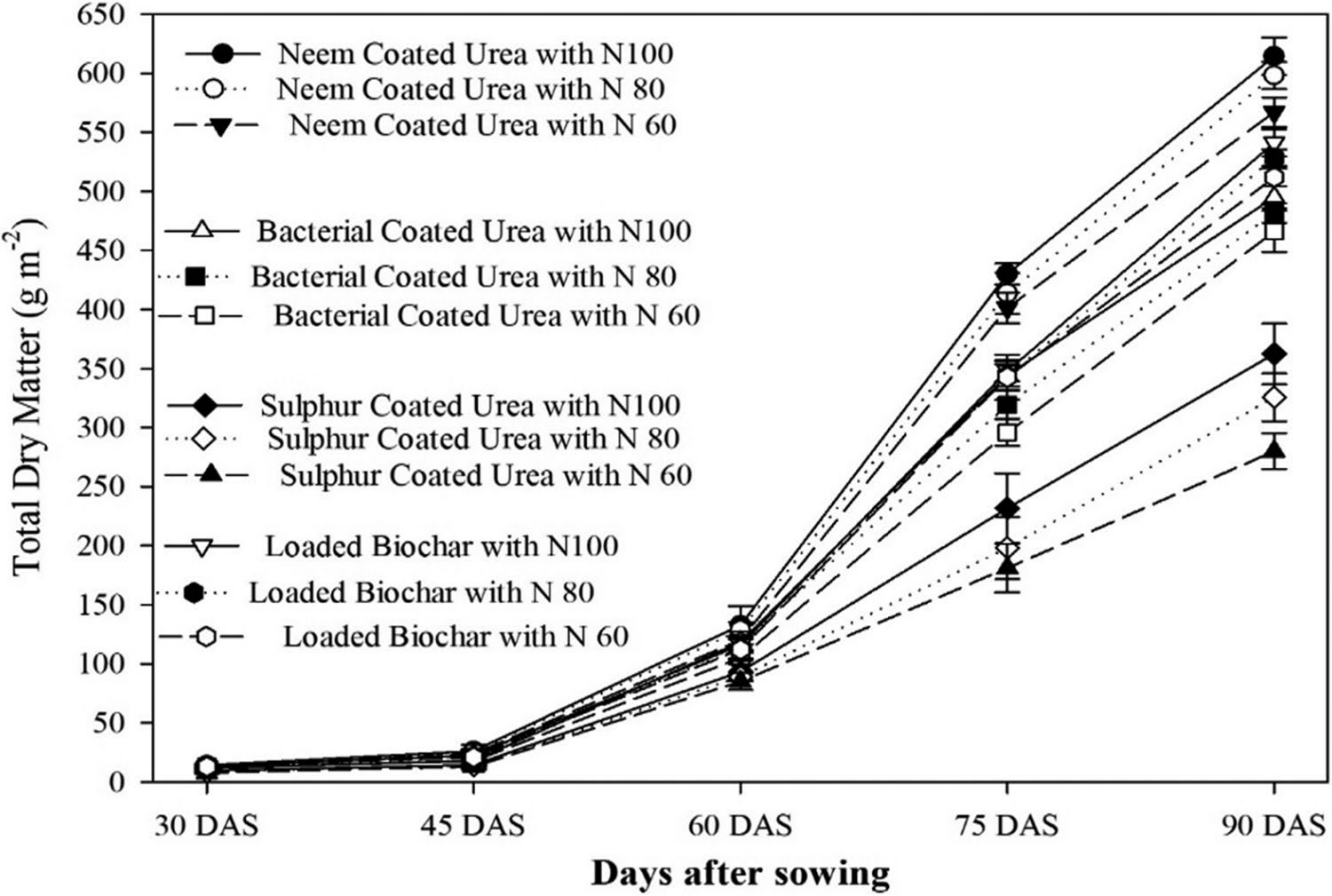
Effect of slow release nitrogen fertilizer (SRNF) and N levels on the total dry matter accumulation at different critical growth stages of sunflower crop under arid climatic conditions

The results indicated that the effects of different SRNF and N increments were found significant on growth and development parameters (leaf area index, crop growth rate, and total dry matter) (Fig. 3). Maximum LAI (2.12, 3.62, 5.97, and 3.00) were observed at 45, 60, 75, and 90 DAS when the crop was fertilized with neem-coated urea 100% (148 kg N ha^−1^) that was statistically same with application of loaded biochar 100% of recommended N (46% N + BC 2 tons ha^−1^). The maximum CGR was also observed (19.16 g m^−2^d^−1^) with neem-coated urea 100% of recommended (148 kg N ha^−1^) application at 60–75 DAS that was statistically similar, when sunflower was fertilized with loaded biochar 100% of recommended (46% N + BC 2 tons ha^−1^) which produced crop growth rate of 15.67 g m^−2^d^−1^ (Fig. 3). Furthermore, CGR at 45 DAS rapidly increase and gain maximum at 60–75 DAS, and then CGR starts declining after 75 DAS, and the minimum was recorded during 75–90 DAS. Neem-coated urea with 100% of recommended (148 kg N ha^−1^) dose increase total dry biomass significantly. Maximum dry biomass (14.27, 26.29, 122.67, 410.00, and 604.33 g m^−2^) was noted at 30, 45, 60, 75, and 90 days after sowing. Neem-coated urea that was statistically at par when crop was fertilized with loaded biochar 100% of recommended (46% N + BC 2 tons ha^−1^) which produced dry biomass (12.97, 22.78, 116.00, 348.00, and 541.00 g m^−2^) at 30, 45, 60, 75, and 90 days after sowing (Fig. 4).

### Yield and yield contributing attributes of sunflower under arid climatic conditions

The results of current study revealed that different SRNF and N levels significantly affected the yield-related attributes of the sunflower crop (mean plant height, diameter and achenes per head, weight of 1000-achene, sunflower achene, and biological yield and harvest index) at probability level (*p*) ≤ 0.05 (Table 2). Furthermore, the interactive effect of various SRNF with N levels was not found significant for all the responsive yield-related attributes at *p* ≤ 0.05 (Table 2). Sunflower crop showed the highest plant height (198 cm) when it was fertilized with N increment N_100_ (148 kg ha^−1^) in comparison to N increments N_80_ (118 kg ha^−1^) and N_60_ (89 kg ha^−1^) (Table 2). Sunflower crop showed the highest head diameter (18.7 cm) with the application of loaded biochar which was at par when sunflower crop was fertilized with the bacterial coated urea and neem-coated urea (Table 2). Sunflower crop showed the highest achenes per head (1278) with neem-coated urea which was at par bacterial coated urea and loaded biochar fertilization. Moreover, sunflower crop fertilized with (N) increment N_100_ (148 kg ha^−1^) showed highest achene per head (1455) (Table 2). Sunflower crop showed highest 100-achene weight (55 g) with neem-coated urea fertilization, and it was at par with the bacterial coated urea and loaded biochar fertilization. Furthermore, when sunflower crop fertilized with (N) increment N_100_ (148 kg ha^−1^) showed highest 1000-achene weight (56.3 g) (Table 2). Moreover, sunflower crop showed maximum achene yield (2322 kg ha^−1^) with neem-coated urea fertilization which was at par when sunflower crop was fertilized with the loaded biochar and bacterial coated urea. Likewise, when sunflower crop fertilized with (N) increment N_100_ (148 kg ha^−1^) showed also maximum achene yield (2469 kg ha^−1^) (Table 2). Sunflower crop showed highest biological yield (9000 kg ha^−1^) with neem-coated urea which was at par when sunflower crop was fertilized with the loaded biochar. Likewise, when sunflower crop fertilized with (N) increment N_100_ (148 kg ha^−1^) showed highest biological yield (9211 kg ha^−1^) (Table 2). Sunflower crop showed maximum harvest index (25.8%) with neem-coated urea fertilization which was at par when sunflower crop was fertilized with the loaded biochar and bacterial coated urea. Furthermore, when sunflower crop fertilized with (N) increment N_100_ (148 kg ha^−1^) showed maximum harvest index (26.8%) (Table 2).

**Table 2:** Effect of different SRNF and N levels on the yield-related parameters of sunflower crop under arid climatic conditions

Treatments	Plant height (cm)	Head diameter (cm)	Achenes per head	1000-achene weight (g)	Achene yield (kg ha^−1^)	Biological yield (kg ha^−1^)	Harvest index (%)
Slow release (N) fertilizers							
Neem-coated urea	191	18.2 ab	1278 a	55.0 a	2322 a	9000 a	25.8 a
Bacterial coated urea	190	17.8 ab	1219 a	54.2 bc	2269 a	8942 ab	25.3 a
Sulfur-coated urea	190	17.4 b	1072 b	53.9 c	2101 b	8818 b	23.8 b
Loaded biochar	190	18.7 a	1235 a	54.7 ab	2283 a	8981 a	25.3 a
LSD (*p* ≤ 0.05)	–-	1.04	133	0.67	155	137	1.39
N increments							
N_100_ = 148 kg ha^−1^	198 a	18.8	1455 a	56.3 a	2469 a	9211 a	26.8 a
N_80_ = 118 kg ha^−1^	187 b	18.0	1188 b	54.3 b	2270 b	8935 b	25.4 b
N_60_ = 89 kg ha^−1^	185 c	17.3	959 c	52.7 c	1993 c	8660 c	23.0 c
LSD (*p* ≤ 0.05)	1.02	–-	115	0.58	134	119	1.20
SRNF	NS	**	**	**	**	**	**
NI	**	NS	*	**	*	*	*
SRNF × NI	NS	NS	NS	NS	NS	NS	NS

*SRNF* slow release (N) fertilizers; *NI* (N) increments; **significant at *p* ≤ 0.01; *significant at *p* ≤ 0.05; *NS* non-significant at *p* ≤ 0.05.

### Nitrogen use efficiency and soil available (N) at different soil depths

**Fig. 5:**
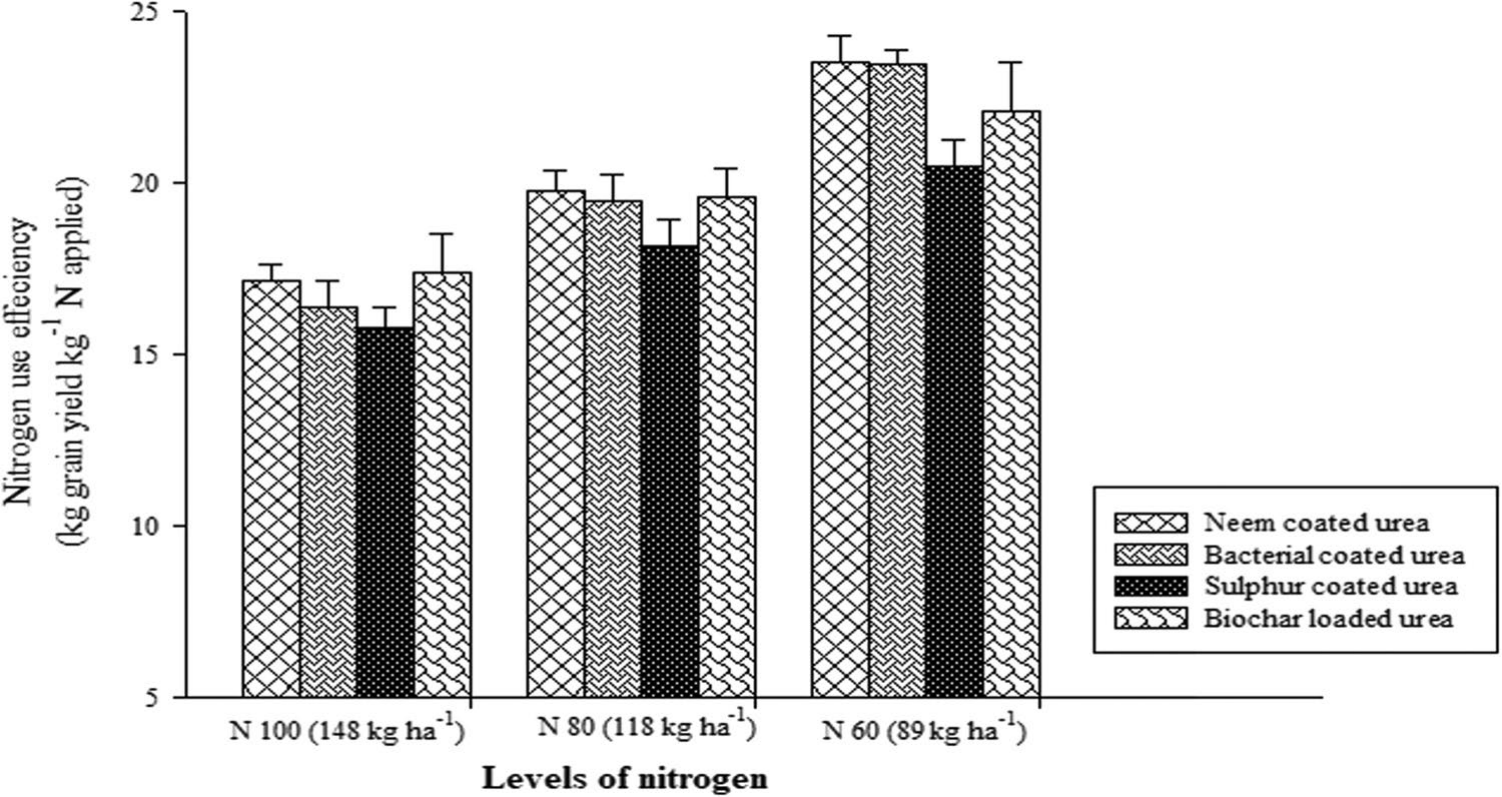
Effect of different SRNF and N increments on NUE of sunflower crop under arid climatic conditions

**Fig. 6:**
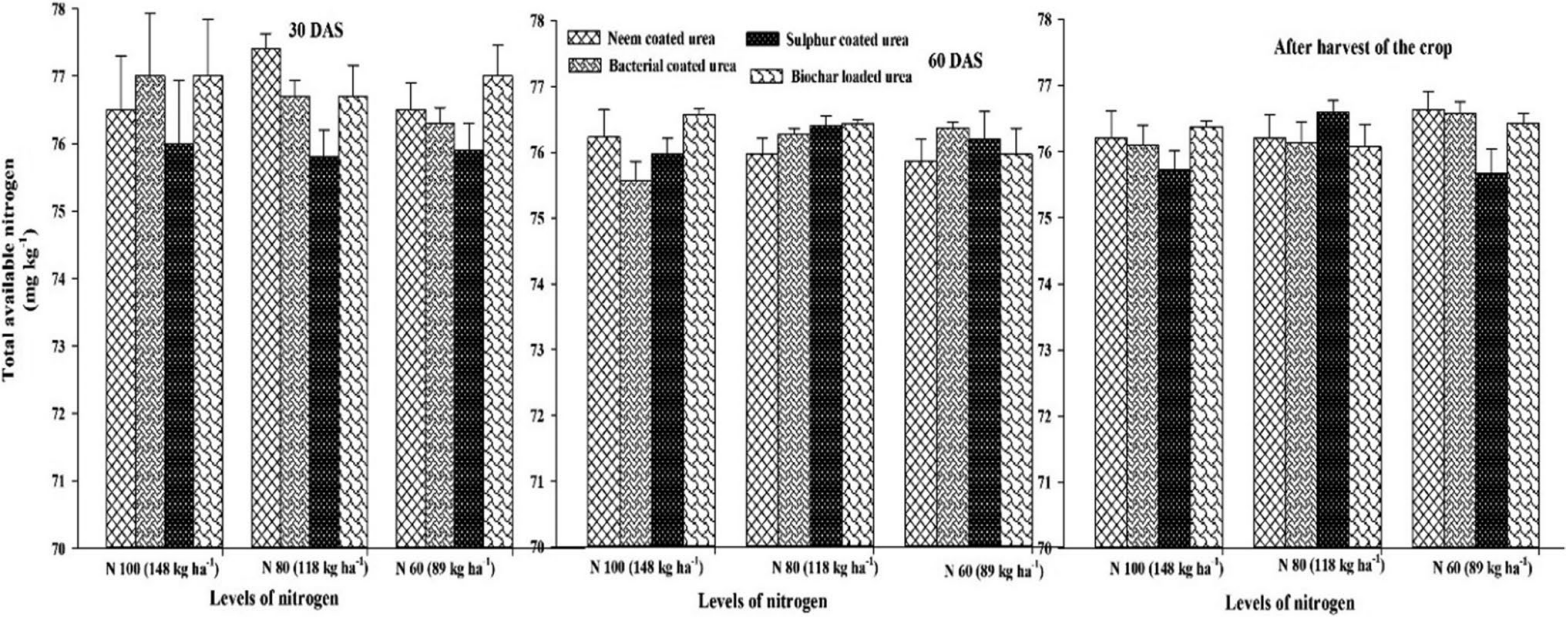
Effect of different SRNF and N levels on soil available N at 0–15 cm after 30 DAS and 60 DAS and at harvest stage of sunflower crop

**Fig. 7:**
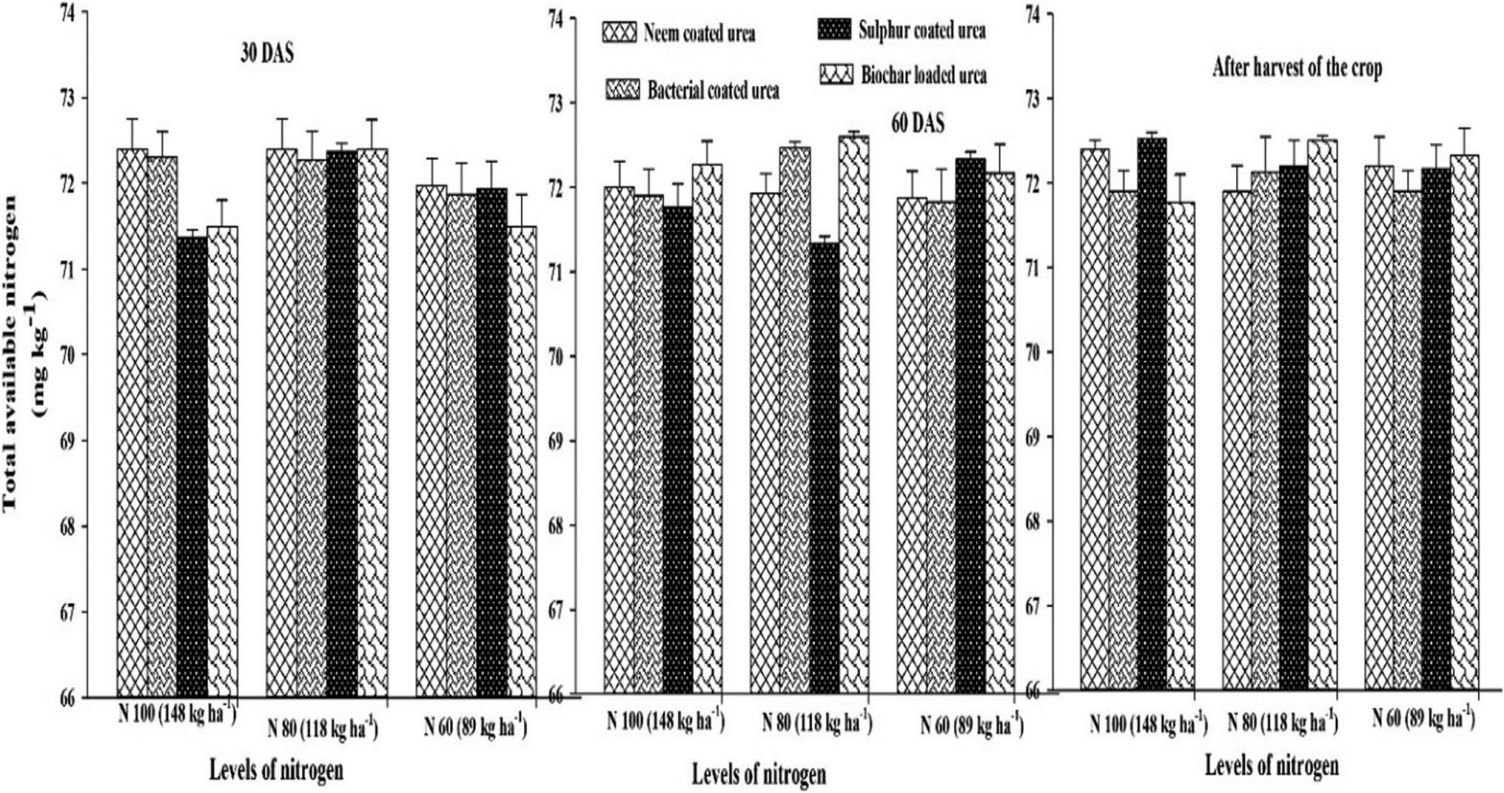
Effect of different SRNF and N increments on soil available (N) at 15–30 cm after 30 DAS and 60 DAS and at harvest stage of sunflower crop

**Fig. 8:**
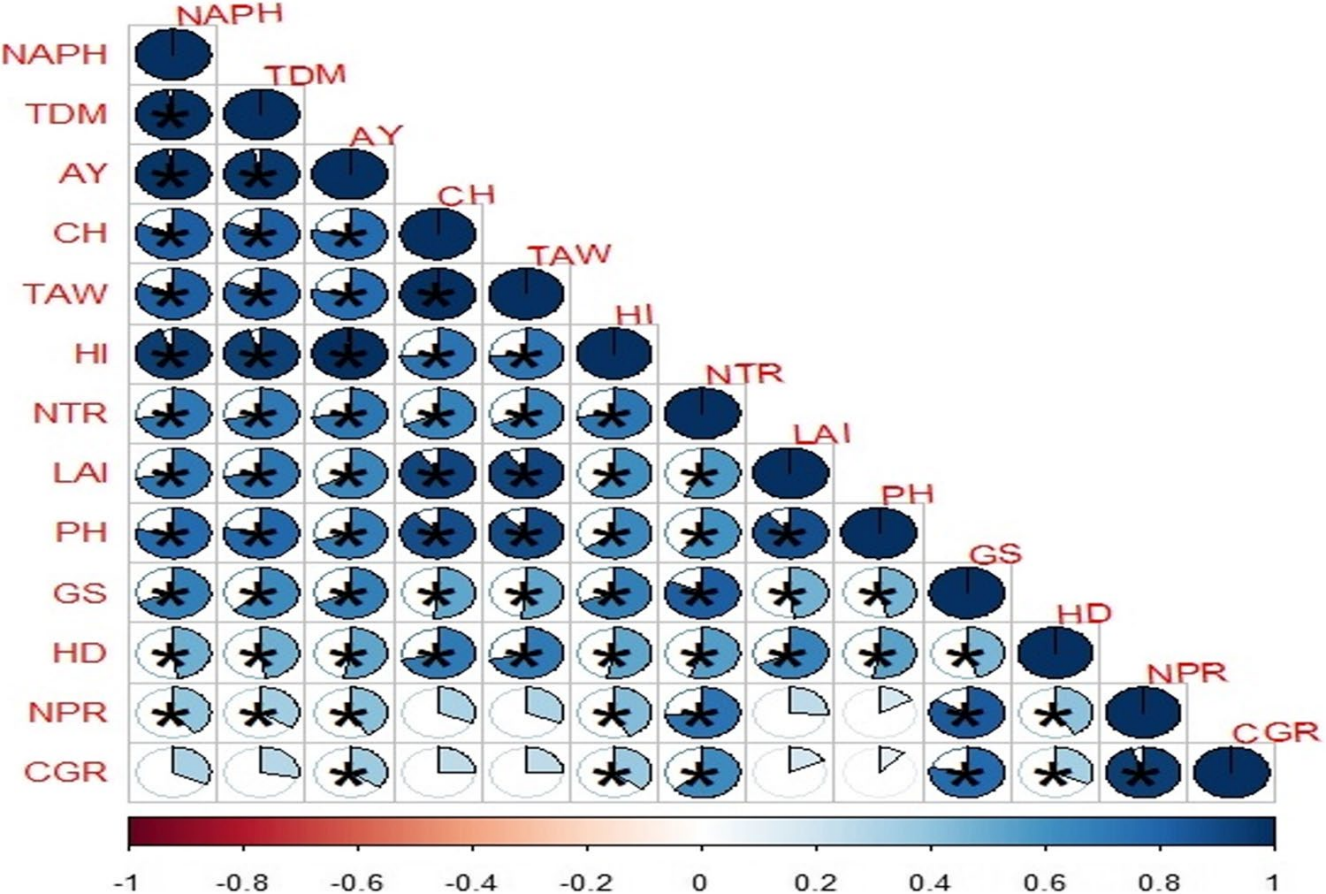
Correlation map of different traits (growth, physiology, and yield-related traits) of sunflower crop under different SRNF and N levels. Highlighted circled areas specify absolute quantity of related correlation coefficients at *p* ≤ 0.05 significance level

The results of the current field study indicated that different SRNF and N increments markedly affected (N) use efficiency (NUE) of sunflower crop. However, interactive effect of different SRNF with N increments was non-markedly effective for NUE of the sunflower crop (Fig. 5). The maximum NUE was noted when fertilized with neem-coated urea which was statistically similar with NUE obtained by the application of bacterial coated urea and loaded biochar. However, minimum NUE was noted sulfur-coated urea fertilization. Among various N increments, increment N_60_ produced maximum NUE of sunflower crop. However, N increment N_100_ (148 kg ha^−1^) produced minimum NUE of sunflower crop (Fig. 5). This study showed that various SRNF and N increments non-markedly affected the soil available (N) at 0–15 and 15–30 cm depths (Figs. 6, 7). Similarly, two-way interaction of various slow release (N) sources and (N) increments did not markedly affect the soil available (N) at 0–15 and 15–30 cm (Figs. 6, 7). As represented in Fig. 3, the crop growth rate (CGR) is significantly different with the net photosynthetic rate (NPR), while the minimum difference was found for plant height (PH). Net photosynthetic rate relation was significantly related to the stomatal conductance (GS) value, while same as crop growth rate, it was less related to the plant height value. This correlation explains that harvest index (HI) has the same relationship with the values of leaf area index (LAI), 1000-achene weight (TAW), and chlorophyll contents as compared to stomatal conductance value. In all the correlations, there was a significant difference (Fig. 8).

## Discussion

Nitrogen is an imperative macronutrient that is demanded in large amounts for sunflower production (Nasim et al. 2017). But nitrogen deficiency and low nitrogen use efficiency negatively effects the sunflower growth and yield (Ahmad et al. 2018). In the current study the LAI, CGR and time series TDM were highest with neem-coated urea fertilization at N increment N100 (148 kg ha^−1^) in comparison to all other SRNF at N increment N100 (148 kg ha^−1^). These results were correlated with higher availability of nitrogen and imperious role of N in improving up all the plant functions by the production of protein, enzymes, hormones, chlorophyll, and vitamins which ultimately resulted in higher LAI, CGR, and time series TDM. Furthermore, higher values of LAI, CGR, and time series TDM were because of the more availability of (N) by reducing the losses of (N) in the form of runoff, nitrate leaching, and ammonia volatilization; hence, continuous availability of (N) due to the slow release of (N) from neem-coated urea. The increase in LAI was attributed due to the more expansion of leaves as the plants were using their whole (N) requirement that is why the growth of these plants treated with coated fertilizers was more effective. With this factor, all the other growth parameters were also enhanced as these are correlated with each other (Hassan et al. 2021; Li et al. 2018; Wajid et al. 2012). Moreover, the CGR increases rapidly with the application of non-coated behavior in the first two and half months. But, after that, growth of the crop slows down in the third month, and ultimately decline started during the end of the third month (Nasim et al. 2011). A similar investigation has found that application of slow release fertilizers and biochar sources promotes the growth and development of sunflower significantly and enhances the nitrogen (N) supply in the whole growth periods; it is because of lesser N losses in the different forms (Wang et al. 2013; Javeed et al. 2021; Perveen et al. 2021). Similarly, higher values of net leaf photosynthetic rates, net transpiration rates, stomatal conductance, and chlorophyll content might be due to the slow and successive discharge of N from neem-coated urea (Ghafoor et al. 2021) which corresponded well to the N requirements at critical growth stages of sunflower. The increase in net photosynthetic rate is may be due to the more chlorophyll contents of the sunflower leaves as the more production of proteins supports the increase in leaves area and chlorophyll contents as well. Similarly, the more leaf size and more leaf area will have more number of stomata which are the main contributors of the transpiration in plants. Stomatal conductance also increases with the better growth of the sunflower plants (Glass, 2003; Ahmad et al. 2018).

An increase in the growth parameters will also cause an increase in the yield contribution as all the growth parameters are directly related to the production of yield components. Plant height, head diameter, 1000-achene weight, achenes per head, achene yield, biological yield, and harvest index were significantly affected with the application of neem-coated urea at N increment of N100 (148 kg ha^−1^). These results might be owing to maximum N availability, and it might be substantially supported due to quick roots and leaves growth and chlorophyll synthesis which resulted in maximum biomass accumulation and ultimately higher plant height, head diameter, 1000-achene weight, achene yield, biological yield, achenes per head, and harvest index of sunflower crop. More biomass production is an indication of more yield and coated fertilizers help in increasing biomass production as compared to those with non-coated fertilizers (Jin et al., 2010). Higher N fertilization has a direct important influence on the 1000-achene weight shown in Table 1 which can be assessed through dry matter attained to sunflower heads from the flowering parts (Gardner et al. 1994). Moreover, it proves that stem has a vital role as storage source of carried carbohydrates, and it mobilizes carbohydrates to flowering and physiological maturity as well. It is due to excess N availability and enhanced both productive and reproductive parts which resulted in maximum diameter of sunflower heads and 1000-achene weight because of more nutrient availability. For the better growth and development of plants, higher utilization of the available resources like fertilizer are also correlated with the ideal growing conditions. This will not only flourish the growth of plants but also cause a better reward in case of achene yield improvement (Zahoor et al. 2010; Wajid et al. 2010; Zubillaga et al. 2002). Among various yield-related traits (achenes per head and sunflower achene yield), different SRNF and N levels were significant among each other as shown in Table 2. Several investigations had verified results that improvement in NUE enhances CGR, LAI, 1000-achene weight, and head diameter and ultimately markedly enhances achene yield as shown in Fig. 3, and Table 2 and similar findings have also been previously reported (Abbadi and Gerendas 2009; Wajid et al. 2010). Previous studies cleared the enhanced achene yield in sunflower due to N fertilizer and low achene yield as a result of less yield of N fertilizer (De La Vega and Hall 2002; Tovar et al. 2021). This study showed that high nitrogen with neem-coated source obtained maximum harvest index in comparison to low N applied and coating with other nitrogen sources (Table 2). Markedly high harvest index for sunflower might be attained due to appropriate availability of N by using coated sources (Jin et al. 2010; Koutroubas et al. 2008). The gap between highest and lowest harvest index of sunflower crop attained at different SRNF and N levels was statistically significant as shown in Table 2. The effects of SRNF showed improvements in the studied yield components because of slowly release of N from SRNF which matched well to the demand of sunflower crop. High NUE is directly related to less N losses like leaching, volatilization, runoff, and denitrification (Nasim et al. 2017). Nitrogen use efficiency was observed maximum with the use of slow release fertilizer as compared to simple urea (Geng et al. 2016). Among all the N sources application, slow release and coated fertilizers are the best N sources as these sources provide the more accurate amount of nitrogen at the time of crop requirement with lower losses in the soil and environment.

## Conclusions

In the current study, different nitrogen increments significantly affected growth, physiology, and yield-related traits of sunflower crop. Similarly, slow release nitrogenous fertilizers significantly affected growth, physiology, and yield-related attributes and N use efficiency.. Neem-coated urea showed higher growth, yield, and yield attributes of sunflower in comparison to simple urea and slow release nitrogenous fertilizers. Furthermore, nitrogen increment N_100_ (148 kg ha^−1^ N) also showed growth, yield, and yield attributes and N use efficiency of sunflower in comparison to other N increments. In crux, neem-coated urea with recommended N (148 kg ha^−1^) and 80% of recommended N application (118 kg ha^−1^) would be recommended to enhance the achene yield and the NUE in the sunflower production system in the region. Future studies are also needed to evaluate the response of various N increments and N sources on sunflower growth, physiology, and yield-related parameters across the contrasting environments, and further modeling studies may also require to assess the impact and losses under future climate scenarios of the region for sustainable production.

## Data Availability

The datasets and codes used and/or analyzed during the current study are available from the corresponding author on reasonable request.
